# Validation of a single-platform, volumetric, flow cytometry for CD4 T cell count monitoring in therapeutic mobile unit

**DOI:** 10.1186/1479-5876-10-22

**Published:** 2012-02-06

**Authors:** François-Xavier Mbopi-Kéou, Bertrand Sagnia, Jeanne Ngogang, Fru F Angwafo III, Vittorio Colizzi, Luc Montagnier, Laurent Bélec

**Affiliations:** 1 Laboratoire National de Santé Hygiène Mobile, Ministère de la Santé Publique, and Faculté de Médecine et des Sciences Biomédicales, Université de Yaoundé I, Yaoundé, Cameroun; 2 Centre International de Référence Chantal Biya, Yaoundé, Cameroun; 3 Centre Hospitalier-Universitaire de Yaoundé, and Faculté de Médecine et des Sciences Biomédicales, Université de Yaoundé I, Yaoundé, Cameroun; 4 Laboratoire de Virologie, Hôpital Européen Georges Pompidou, and Faculté de Médecine Paris Descartes, Université Paris Descartes (Paris V), Sorbonne Paris Cité, Paris, France

**Keywords:** Flow cytometry, CD4 T cell count, HIV, Resource-limited settings, Therapeutic mobile unit

## Abstract

**Background:**

A mobile health unit may be useful to follow up adult and pediatric patients on antiretroviral treatment and living in remote areas devoid of laboratory facilities. The study evaluated the use of the simplified, robust, single-plateform, volumetric, pan-leucogating Auto40 flow cytometer (Apogee Flow Systems Ltd, Hemel Hempstead, UK) for CD4 T cell numeration in a mobile unit, compared against a reference flow cytometry method.

**Methods:**

The therapeutic mobile unit of the Laboratoire National de Santé Hygiène Mobile, Yaoundé, Cameroon, was equipped with the Auto40. A FACSCalibur flow cytometer (Becton Dickinson Immuno-cytometry System, San Jose, CA, USA) was used as reference method. EDTA-blood samples from volunteers were first subjected to CD4 T cell count in the mobile unit, and an aliquot was sent within 4 hours to Centre International de Référence Chantal Biya, Yaoundé, for FACSCalibur assay.

**Results:**

Two HIV screening campaigns with the mobile unit were organised in December 2009 and January 2010. The campaign in the suburb of Yaoundé which was 20 km from the reference laboratory included 188 volunteers comprising 93 children less than 5 years old. The campaign in Ambang Bikok (53 km far from Yaoundé) included 69 adult volunteers. In Yaoundé suburb, mean ± standard deviation (SD) CD4 T cell count was 996 ± 874 cells/μl by Auto40, and 989 ± 883 cells/μl by FACSCalibur; in Ambang Bikok, mean ± SD CD4 T cell count was 1041 ± 317 cells/μl by Auto40, and 1032 ± 294 cells/μl by FACSCalibur. Results by Auto40 and FACSCalibur were highly correlated in Yaoundé (r^2 ^= 0.982) as in Ambang Bikok (r^2 ^= 0.921). Bland-Altman analysis showed a close agreement between Auto40 and FACSCalibur results expressed in absolute count as in percentage in Yaoundé and Ambang Bikok. When pooling the 257 CD4 T cell count measurements, the Auto40 yielded a mean difference of +7.6 CD4 T cells/μl higher than by reference flow cytometry; and the sensitivity and specificity of Auto40 in enumerating absolute CD4 T cell counts of less than 200 cells/μl were 87% and 99%, respectively, and in enumerating absolute CD4 T cell counts of less than 350 cells/μl were 87% and 98%, respectively. The intrarun and interun precisions of the Auto40 assay assessed in the mobile unit were 5.5% and 7.9%, respectively.

**Conclusions:**

The Auto40 flow cytometer installed in a therapeutic mobile unit and operated far from its reference laboratory gave a perfect correlation with the reference method, and could be useful in carrying out immunological monitoring of HIV-infected patients living in areas without access to laboratory facilities.

## Background

The 2010-revised guidelines of the World Health Organization (WHO) for scaling up of antiretroviral treatment (ART) in adults and children living in resource-limited settings [1,2], emphasizes the need of laboratory monitoring, based first on immunological assessment by the numeration of CD4 T lymphocytes, mainly to start ART and monitor patients on ART, and secondly on HIV-1 RNA load in order to monitor treatment efficacy, early therapeutic failure and subsequent therapeutic switch [3-6]. Affordable CD4 T cell counting has gradually become possible by using simple, compact and robust low-cost new generation cytometers operating as single-platform volumetric instruments without the use of expensive micro beads [7-11].

The scaling up of public ART programs globally has led to an increased demand for CD4 T cell count tests [12], especially to assess treatment eligibility. CD4 T cell count has become the most decentralized and universally available biological marker to monitor HIV disease and ART [3]. However, access to CD4 T cell measurement remains a bottleneck to ART scale-up and decentralization, especially in remote rural areas frequently lacking laboratory infrastructure [13,14].

In order to increase the number of individuals tested for HIV, in keeping with our target of universal access, we recently developed a strategy based on bringing the healthcare package much closer to the people, through mobile HIV testing units [15], conceived to bring education and testing services to hard-to-reach population [16]. This pilot study, the first in the Central African region, clearly demonstrated the acceptability, feasibility and effectiveness of using mobile units as a tool for mass HIV testing in individuals with limited access to voluntary counseling and testing [15]. Successful HIV testing with mobile units was unexpected, given that resistance and sociocultural impediments to HIV testing have been identified in Central Africa [17]. In addition, previous successful experiences in South Africa [18] and Kenya [19] showed that mobile services for HIV screening are accessed by different target population compared with facility-based services, including younger people, and higher proportion of persons with newly diagnosed HIV infection.

HIV counseling and screening mobile units may be complemented with antiretroviral drugs and laboratory facilities to become ambulatory health units for ART and follow up adult and pediatric patients living in remote areas devoid of laboratory facilities. The recently developed Auto40 flow cytometer (Apogee Flow Systems Ltd, Hemel Hempstead, UK; http://www.ApogeeFlow.com) was originally developed for military applications [10]. It has been adapted for CD4 T cells measurement within 30 minutes, using a pan-leucogating protocol with anti-CD4 and anti-CD45 thermo-stable monoclonal antibodies. Due to the stability of its optical bench, the Auto40 has been conceived for use on mobile or peripheral stationary flow cytometry unit [10]. Finally, the aim of the present study was to evaluate the usefulness of the simplified Auto40 flow cytometry system for CD4 T cell numeration in mobile health unit, according to a reference flow cytometry method.

## Materials and methods

### Volunteers, blood sampling and processing

In December 2009 and in January 2010, two HIV screening campaigns with mobile units were organized in the outskirts of Yaoundé, the capital city of Cameroon, 20 km away from the Laboratoire National de Santé Hygiène Mobile (LNSHM), the reference laboratory for HIV screening mobile campaign in Cameroon [15], and in Ambang Bikok, a rural setting located 53 km from Yaoundé. In a suburb of Yaoundé, children followed up at the Nkoldongo Medical Health District were also included for CD4 T cell measurement, as part of an agreement with the Chantal Biya International Reference Centre for Research and Prevention of HIV/AIDS (CIRCB), Yaoundé, for free HIV biological monitoring. Volunteers had access to primary health care centers and antenatal clinics, but modern laboratory facilities were not routinely available.

An aliquot of K3-EDTA-blood sample obtained by venipuncture in Vacutainer tubes (Becton Dickinson, Franklin Lakes, NJ, USA), kept at ambient temperature, was obtained after informed consent from each volunteer, patient or child guardian. Each aliquot was first subjected to CD4 T cell count in the therapeutic mobile unit, and a second aliquot was send within 4 hours at CIRCB for measurement by flow cytometry reference analyzer in the same day of sampling. All blood samples were unlinked to identifiers.

### CD4 T cell measurements

CD4 T cell counting was performed on 2 different flow cytometers: (1) the FACSCalibur [Becton Dickinson Immuno-cytometry System (BDIS), San Jose, CA, USA], a dedicated clinical instrument for CD4 T cell counting installed at the CIRCB, used as reference method, and (2) the Auto40 flow cytometer (Apogee Flow Systems Ltd) equipped with a green laser at 532 nm, a side scatter detector, two fluorescence channels and means for direct volumetric counting, without requiring a step of red blood cell lysis. The Auto40 flow cytometer has been installed in the therapeutic mobile unit of the LNSHM, using the battery of the van as the source of electrical energy. An optional sheath fluid recycling cassette reduces sheath fluid consumption and may be of interest in remote areas where the supply is limited.

The FACSCalibur was used as predicate instrument for CD4 T cell counting in the study, as a single-platform flow cytometry technique. Absolute CD4 T cell counting was performed according to the instructions provided by the manufacturer. In brief, 50 μl of EDTA-blood and 10 μl of Multitest reagent (BDIS) were added to the dedicated TruCOUNT™ tubes (BDIS) containing a fixed number of polystyrene reference beads and then vortexed and left to incubate for 15 minutes at room temperature. The Multitest reagent is combined with four fluorescent [fluorescein isothiocyanate (FITC), phycoerythrin (PE), peridinin chlorophyll protein (PerCP), and alophycocianin (APC)]-conjugated monoclonal antibodies reagents for CD3/CD8/CD45/CD4 (FITC-CD3/PE-CD8/PerCP-CD45/APC-CD4). Red blood cells were then lysed by using FACS Lysing Solution (BDIS). The lyse-no-wash stained samples were run on the FACSCalibur. Data were analyzed using MultiSet V2.2 software (BDIS) for calculating the absolute and percentage values of CD4 T cells. The CD8 T cell counting allowed by the Multitest reagent was not used in the present study

The Auto40 assay is based on the no lyse procedure [20], which avoids the red blood cell lysis step, thereby reducing assay variability due to changes in assay conditions (time and temperature of incubation) as well as differences in the susceptibility of cells to the lysis reagents [21]. The Auto40 analyzer uses a volumetric syringe moved by a stepper motor that draws and delivers a known sample volume. Therefore, its absolute volumetric counting allows the direct determination of the number of cells per unit of sample volume without the need for reference material such as micro beads [8,22]. Thus, direct volumetric CD4 T cell measurements were performed on the Auto40 using PE-conjugated anti-CD4 and PE-Dyomics649-conjugated anti-CD45 monoclonal antibodies (Apogee Flow Systems Ltd) for CD4 T cell count measurement. Anti-CD4 and anti-CD45 monoclonal antibodies are resistant to high temperatures because of their stabilization by a special dehydration process under controlled conditions (proprietary procedure, Bio-D) [11]. The Auto40 analytical procedure avoids the need for a wash step. Briefly, 50 μl of whole EDTA-blood was added into polypropylene test tubes containing pre-dispensed, stabilized monoclonal antibodies. After 25 minutes of incubation at room temperature in the dark, 450 μl of phosphate buffered saline were added. The no-lyse-no-wash stained samples were run on the Auto40 flow cytometer, and CD4 T cell count was obtained in absolute number and in percentage. Analysis on the Auto40 flow cytometer is automatically performed by the built-in very friendly software "Auto-Lymphocyte" (Apogee Flow Systems Ltd), with the possibility of controlling and assessing the quality of the data analysis, particularly when the CD4 T cell number is less than 40 CD4 T cells/μl. Aside from simplifying sample preparation, this system greatly facilitates manipulations by lab technicians. The training of technicians to use the Auto40 analyzer takes roughly one day.

### Assessment of the precision of CD4 T cell counting in the mobile unit

The precision of CD4 T cell counting on the Auto40 instrument in the mobile unit was assessed using 2 different blood samples. Intrarun (instrument) precision was determined by preparing a large volume (10 ml) of CD4-stained blood and repeating sample acquisition on the instrument at least 10 times to determine the instrument's precision. Interrun precision, which includes the variation induced by pipetting errors made by the technician (tube-to-tube variability), was assessed by repeating the entire CD4 staining procedure at least 10 times: pipetting, sample preparation, staining, and sample acquisition. By comparing the intrarun and interrun precision, a fairly good impression of instrument's and technician's performance can be obtained. Interperson variation (between different technicians) was not assessed. Precision was expressed as the coefficient of variance (CV) obtained by dividing the standard deviation (SD) of all the measurements by the mean (CV% = SD × 100/mean).

### Statistical analyses

The Method Validator software, version 1.1.9.0. (Philippe Marquis, France) and the SAS-PC software (version 8.2, SAS Institute, Cary, North Carolina, USA) were used for analyses. Firstly, correlations between the absolute CD4 T cell counts obtained by the reference FACSCalibur and the mobile Auto40 were established by the Passing-Bablok method which, in common with all nonparametric methods, is less sensitive to outliers [23]. Secondly, the agreement between the two methods was illustrated by difference plots as proposed by Bland and Altman [24,25]. The average difference between the 2 methods, referred to as bias, was marked on the graph by a horizontal line, and the mean difference and the limits of agreement with a 95% confidence interval (CI) were also depicted.

To assess the clinical impact of using the Auto40 instead of the FACSCalibur in this setting, the sensitivity and the specificity of the Auto40 was calculated to identify patients who had with the FACSCalibur a CD4 T cell count below 200 cells/μl the threshold of immune-restoration under ART and the threshold for therapeutic initiation according to the 2006-revised WHO recommendations [26] or 350 cells/μl, the new threshold for ART initiation according to the 2010-revised WHO guidelines [1]. For clinical significance of the measurement differences on treatment decision, the Cohen's *k *coefficient was calculated on the whole study population [27].

## Results

### Accuracy of direct volumetric CD4 T cell measurements on the Auto40 using EDTA-containing whole blood

Study volunteers were included irrespective of their HIV sero-status (except children). The campaign in the suburb of Yaoundé included 98 adults volunteers (median age, 39.5 years; 28 males) for CD4 T cell count, and 93 children less than 5 years and more than 18 months (43 males). The campaign in Ambang Bikok included 69 adult volunteers (median age, 34 years; 39 males). Three blood samples from adult volunteers showed poor pre-analytical preparation when arriving at CIRCB, and were excluded from further analysis. Finally, parallel CD4 T cell measurements on both instruments, the Auto40 and FACSCalibur, were available for a total of 257 tested blood samples (Table 1).

**Table 1:** CD4 T cell counting in Yaoundé suburb and Ambang Bikok village obtained by the Auto40 flow cytometer installed in a therapeutic mobile unit, and by the FACSCalibur at the reference laboratory, expressed in 5^th^-95^th ^percentile and mean.

	n*	5^th^- 95^th ^percentile	Mean ± SD	P value**
**Yaoundé suburb**	188			
. Absolute CD4 T cells***				
- Auto40		111-2624	996 ± 874	
- FACSCalibur		110-2620	989 ± 883	NS
. Percent CD4 T cells				
- Auto40		6.0-48.0	27.1 ± 13.6	
- FACSCalibur		6.0-49.0	36.7 ± 13.1	NS
				
**Ambang Bikok**	69			
. Absolute CD4 T cells				
- Auto40		530-1585	1041 ± 317	
- FACSCalibur		585-1511	1032 ± 294	NS
. Percent CD4 T cell				
- Auto40		26.8-52.0	38.5 ± 7.9	
- FACSCalibur		26.2-51.8	37.5 ± 8.0	NS

* Number of blood samples with successful CD4 T cells counting by both Auto40 and FACSCalibur;

** P value for comparison of mean ± SD by Auto40 *versus *FACSCalibur taken as reference;

*** Absolute CD4 T cells are expressed in cells/μl.

SD: Standard deviation; NS: Not significant

In the suburb of Yaoundé, mean ± SD CD4 T cell count was 996 ± 874 cells/μl (range, 5-4295) by Auto40, and 989 ± 883 cells/μl (range, 5-4454) by FACSCalibur. In Ambang Bikok, mean ± SD CD4 T cell count was 1041 ± 317 cells/μl (range, 236-1774) by Auto40, and 1032 ± 294 cells/μl (281-1616) by FACSCalibur. The differences between Auto40 and FACSCAlibur were not statistically significant in suburb of Yaoundé as in Ambang Bikok (P > 0.5).

**Figure 1:**
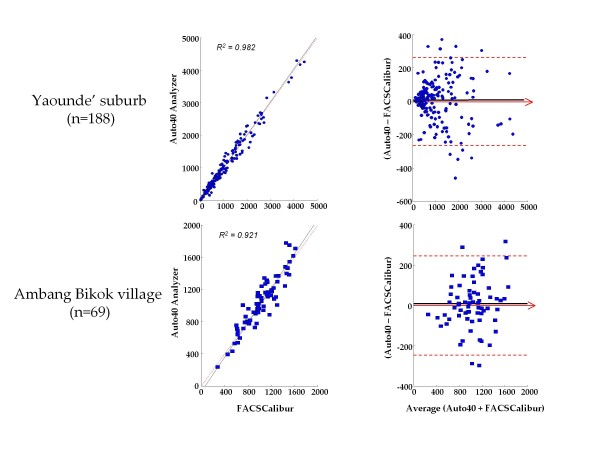
**Results from the campaigns in the suburb of Yaoundé (top) and in the village of Ambang Bikok (bottom), during which 188 and 69 CD4 T cell count measurements, respectively, were carried out in parallel by the Auto40 flow cytometer in mobile unit and by FACSCalibur in the Centre International de Référence Chantal Biya, Yaoundé**. **- Left**. Passing-Bablok agreement test between the CD4 T cell count results expressed in cells/μl obtained with the Auto40 and FACSCalibur. The diagonal dotted line represents the idealline (no bias). The full line represents the regression line of the distribution; **- Right**. Bland-Altman analysis on the relative differences between the absolute CD4 T cell counts obtained with the Auto40 and FACSCalibur flow cytometers compared with the average absolute CD4 T cell count. The black full line represents the mean relative difference, and the other broken lines represent the superior and inferior limits of agreement. The arrow corresponds to the X abscise axis.

Results by Auto40 and FACSCalibur were highly correlated using a non-parametric Passing-Bablok regression analysis in Yaoundé (r^2 ^= 0.98, slope = 1.01, intercept = 8.1) (Figure 1, top) as in Ambang Bikok (r^2 ^= 0.92, slope = 1.07, intercept = -68) (Figure 1, bottom). The relation between Auto40 and FACSCalibur did not differ from linearity in suburb of Yaoundé as in Ambang Bikok (P > 0.1).

In the suburb of Yaoundé, the Auto40 results showed a bias of +6.8 cells/μl (95% CI: -12.4 - 26.0) as compared with FACSCalibur (Figure 1, top). In the village Ambang Bikok, the Auto40 results showed a bias of +9.7 cells/μl (95% CI: -19.9 - 39.4) as compared with FACSCalibur (Figure 1, bottom). Thus, Bland-Altman analysis on the relative differences between the absolute CD4 T cell counts obtained with Auto40 and FACSCalibur with the average absolute CD4 T cell counts results in suburb of Yaoundé and Ambang Bikok showed a close agreement between both methods. When pooling the 257 CD4 T cell count measurements, the Auto40 yielded a mean difference of +7.6 CD4 T cells/μl higher than by reference flow cytometry.

Analysis of CD4 T cell count measurement expressed in percentage showed, similarly to CD4 T cell count expressed in absolute numbers, a high correlation and a close agreement between both CD4 T cell counting methods in Yaoundé as in Ambang Bikok (data not shown).

**Figure 2:**
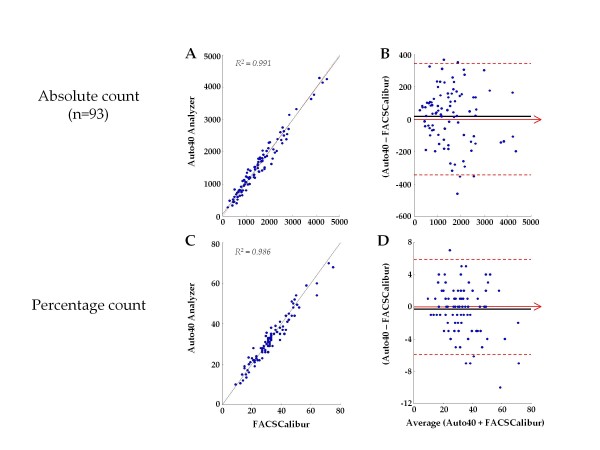
**Results of 93 CD4 T cell count measurements from children aged less than 5 years and more than 18 months in the suburb of Yaoundé, expressed in absolute number (cells/μl) and in percentage carried out in parallel by the Auto40 flow cytometer and by FACSCalibur**. **- A **and **C**. Passing-Bablok agreement test between the CD4 T cell count results obtained with the Auto40 and FACSCalibur flow cytometers, in absolute number (**A**) and in percentage (**C**). The diagonal dotted line represents the ideal line (no bias). The full line represents the regression line of the distribution; **- B **and **D**. Bland-Altman analysis on the relative differences between the CD4 T cell counts obtained with the Auto40 and FACSCalibur flow cytometers compared with the average CD4 T cell count, in absolute number (**B**) and in percentage (**C**). The full line represents the mean relative difference, and the dotted lines represent the superior and inferior limits of agreement. The arrow corresponds to the X abscise axis.

The Figure 2 depicts the results from 93 CD4 T cell count measurements from children aged less than 5 years and more than 18 months, expressed in absolute number and in percentage. In this pediatric population, analysis of CD4 T cell count measurement expressed in percentage showed, similarly to CD4 T cell count expressed in absolute numbers, a high correlation and a close agreement between both CD4 T cell counting methods. Mean ± SD CD4 T cell count in percentage was 32.7 ± 12.1%CD4 (range, 10-70) by Auto40, and 33.0 ± 12.8%CD4 (range, 9-75) by FACSCalibur. The differences between Auto40 and FACSCAlibur were not statistically significant (P > 0.5). Results by Auto40 and FACSCalibur were highly correlated by regression analysis (r^2 ^= 0.986, slope = 1.00, intercept = 0.0) (Figure 2, C). The relation between Auto40 and FACSCalibur did not differ from linearity (P > 0.5). The Auto40 results showed a bias of -0.30%CD4+ (95% CI: -0.91 - 0.31) as compared with FACSCalibur (Figure 2, C), thus demonstrating a close agreement between both methods to measure CD4 T cells in percentage.

### Sensitivity and specificity to identify clinically-relevant thresholds by the Auto40 installed in mobile unit

The sensitivity and specificity of primary CD4 T cell counting on the Auto40 in mobile unit to identify patients having less than (or more than) 200 CD4 T cells/μl was evaluated on the 257 available CD4 T cell count measurements. Taking into account a 10% bilateral range (*i.e*., counts between 190 and 210 CD4 T cells/μl were considered similar), the concordance between the Auto40 and FACSCalibur methods was high (*k *= 0.98; P < 0.01). The decision differed for 2 study individuals. Accordingly, the Auto40 had a sensitivity of 87% and a specificity of 99% to identify individuals with CD4 T cell counts below 200 cells/μl when compared with the FACSCalibur results.

The sensitivity and specificity of primary CD4 T cell counting on the Auto40 in mobile unit to identify patients having less than (or more than) 350 CD4 T cells/μl was also evaluated on the 257 available CD4 T cell count measurements. Considering a 10% bilateral range (*i.e*., counts between 332 and 367 CD4 T cells/μl were considered similar), the decision did not differ between study according to both methods (*k *= 0.96). The Auto40 had a sensitivity of 87% and a specificity of 98% to identify individuals with CD4 T cell counts below 350 cells/μl when compared with the FACSCalibur results.

### Precision of direct volumetric CD4 T cell measurements on the Auto40 in mobile unit

The intra- and inter- run precisions were tested using 2 blood samples. The intrarun precision of Auto40 flow cytometer, expressed as the coefficient of variance, was 3.7% for the first sample (mean ± SD: 425 ± 16 CD4 T cells/μl) and 7.4% for the second sample (122 ± 9 CD4 T cells/μl), giving a mean intrarun precision of 5.5%. The interrun precision performed on the same 2 samples was 6.1% (439 ± 27 CD4 T cells/μl) and 9.7% (133 ± 13 CD4 T cells/μl), respectively, giving a mean interun precision of the Auto40 in mobile unit of 7.9%.

## Discussion

In the present study, we demonstrated that CD4 T cell counting can be integrated into a therapeutic mobile unit and that the Auto40 flow cytometer, whether operated by a laboratory technician, performs acceptably compared with the FACSCalibur for absolute as well percentage count CD4 T cell testing. Thus, the essential WHO recommended ART staging and monitoring diagnostic tests can be accurately conducted at therapeutic mobile unit level using mini flow cytometer to accurately count CD4 T cells. The Auto40 flow cytometer installed in a mobile unit and operated far from its reference laboratory gave a perfect correlation with the reference method. The correlation was maintained over all the dynamic range of values (5-4295 CD4 T cells/μl). CD4 T cell counting in the therapeutic mobile unit permitted identification of the majority of individuals with CD4 T cells below 200 cells/μl, with a sensitivity of 87% and a specificity of 99%, and all individuals with CD4 T cells below 350 cells/μl, with a sensitivity of 87% and a specificity of 98%, demonstrating the capacity of Auto40 in mobile unit to measure in clinical practice CD4 T cells around the threshold of immune-restoration (200 cells/μl), and around the new threshold for therapeutic initiation (350 cells/μl) according to the 2010-revised WHO recommendations [1]. The procedure was fast, and needed only 30 minutes to be completed, with a capacity of 120 tests per day in routine. The technique was found to be easy to carry out and highly reproducible, with intra- and inter- run precisions less than 10%, considered as acceptable for clinical use [10]. Taken together, our observations address the *proof-of-concept *of point-of-care CD4 monitoring by therapeutic mobile unit, and support the decentralization of ART in rural settings with limited laboratory infrastructure. Indeed, the sturdiness of the Auto40 system associated with the use of heat-resistant reagents make possible the immunological monitoring of HIV-infected adults and children living in areas without laboratory facilities.

One of the main features of the Auto40 assay is the use of stabilized monoclonal antibodies. The assay reagents can be stored for prolonged period of time (up to 12 months) at high temperature without any loss of biological activity [11]. This feature is of potential application to the screening of HIV-infected individuals in resource-poor settings where conditions for the storage of reagents, particularly during shipment and delivery, cannot always be guaranteed. The thermo-stable monoclonal antibodies we used in the present study can be kept as long as one year at room temperature, *i.e*. up to +30°C, the stability at higher temperature being unknown. The possibility of long-term storage of reagents at room temperature should facilitate the planning of laboratory activities and reduce the costs related to loss of reagents. Overall, the use of thermo-stable reagents increases the accessibility to flow cytometry testing, since the increase in manufacturing costs related to the antibody stabilization procedure does not exceed 15% of the original cost [11].

The successful scale-up of ART will depend on the ability to deliver health services closer to patients and the community, in primary health care settings [28-30], not only to reach more patients, but also to improve patient outcomes, including higher retention rates [31] and better virological suppression [32-36]. The UNAIDS ART 2.0 model envisions decentralized ART that delivers treatment and care easily and inexpensively, close to where patients live [37]. Decentralization of diagnostics, which are typically conducted at district or central laboratories remote from primary health care settings, is critical to this approach. Patients often make several clinic visits to complete the testing process and results may take days or weeks to return. As transport costs and logistical challenges are significant causes of loss-to-follow-up [38,39], tests conducted closer to the patient may improve retention and treatment success.

A wide assortment of more cost-efficient and technically less complex analyzers using proven flow cytometry-based technology have been developed in response to the overarching challenge of increasing access to CD4 T cell enumeration, and are especially suitable for resource-constrained settings [40]. Affordable CD4 T cell measurement, in absolute number and percentage, has gradually been possible by using simple, compact and robust low-cost new generation flow cytometers operating as single-platform volumetric instruments without the use of expensive micro beads [9]. Currently available mini flow cytometers present convenient efficiency when performed by well-trained laboratory technicians and when combined with good sample transport systems [9,40]. In addition, several other point-of-care CD4 testing options have been conceived, and some are already on the market [41], all in a bid to improve access to CD4 T cell monitoring, especially for rural patients, and to reduce loss-to-follow-up of patients [40,41].

The implementation of CD4 T cell counting in therapeutic mobile unit may help improve access to ART for patients in need, especially for rural populations and populations accessible mainly through mobile and outreach health services [15]. Previous experience on mobile health units for expansion of antiretroviral treatment to remote populations have been reported in Zambia [42]. The mobile service increased the number of ART clients in the study district probably because it reduced the long distances required to travel to health services in rural areas. Interestingly, clients found HIV-infected in mobile unit were generally starting ART at an early stage of HIV disease [42], likely because mobile ART services might have also encouraged people to seek voluntary counseling and testing, much earlier before presenting symptoms. Furthermore, involvement of the community such as lay counselors and support groups increased the number of patients retained at the original site compared to hospitals [42]. Thus, in the Zambian experience, transfers and lost-to-follow-up patients at the mobile sites during the first six months of ART were less frequent, with slightly higher retention rates (70-76%) [42] than generally observed in African hospital-based services (60%) [32]. Although HIV screening in mobile unit was demonstrated to be highly accepted in Cameroonese populations [15], the risk of possible stigma should be always be prevented. Taken together, mobile ART services in resource-limited settings can increase the number of ART patients by reducing the need to travel long distances to reach health facilities, as well as enable patients to start ART at an earlier stage in their disease when HIV diagnosis in made in voluntary counseling and testing center.

Monitoring of CD4 T cell count is cost-effective and may be cost-saving when compared to clinical monitoring alone for determining the timing of ART initiation, way before the development of life-threatening symptoms [43-45]. The issue of whether the monitoring of patients on ART with a mobile unit is a valid economic option remains unresolved. Mobile health clinics are an appealing means of conducting outreach in that they provide an appropriate setting for sexually transmitted disease testing as well as HIV counseling, testing, and referral services. Mobile health vans are expensive, however, frequently costing two to three times more per patient compared with traditional clinics [46,47]. In Uganda, the mobile clinic care for provision of ART has shown to be less cost-effective than facility-based care with higher incremental cost-effectiveness ratio, but would be competitive in targeted "hard-to-reach" population excluded from hospital-fixed health care [48].

## Conclusions

The Auto40 flow cytometer installed in a therapeutic mobile unit and operated far from its reference laboratory gave a perfect correlation with the reference method, could be useful in carrying out immunological monitoring of HIV-infected patients living in areas without access to laboratory facilities, and could likely contribute to the decentralization and scaling-up of ART in Cameroon.

## Competing interests

The authors declare that they have no competing interests.

## Authors' contributions

FXMK, BS, JN, FFA, LB have conceived and designed the research; FXMK, BS have performed the experiments; LB performed statistical analyses; VC, LM help analyzed the results; FXMK, LB drafted the manuscript. All authors read and approved the final manuscript.
